# Inhaled Corticosteroids as an Associated Risk Factor for Asthmatic Pneumonia: A Literature Review

**DOI:** 10.7759/cureus.8717

**Published:** 2020-06-20

**Authors:** Zin Mar Htun, Israa Aldawudi, Prakash C Katwal, Srood Jirjees, Safeera Khan

**Affiliations:** 1 Internal Medicine, California Institute of Behavioral Neurosciences and Psychology, Fairfield, USA; 2 Radiology, California Institute of Behavioral Neurosciences and Psychology, Fairfield, USA; 3 Neurology, California Institute of Behavioral Neurosciences and Psychology, Fairfield, USA

**Keywords:** asthma, inhaled corticosteroids, pneumonia, literature review, budesonide, fluticasone, infection, chronic obstructive pulmonary disease

## Abstract

Asthma patients have commonly been prescribed inhaled corticosteroids (ICSs) as the first line of control therapy. ICSs are associated with an increased risk of pneumonia in chronic obstructive airway disease (COPD) patients. However, the evidence remains controversial in asthma patients. Several observational studies reported an increased risk of pneumonia; however, COPD patients were not excluded clearly in these studies. In observational studies that excluded COPD patients and in randomized controlled trials, ICS use was not found to be associated with the risk of pneumonia. Hence, COPD patients should be excluded in future studies, and the currently available evidence demonstrates that ICS use is not associated with an increased risk of pneumonia in asthma patients.

## Introduction and background

Asthma is a chronic airway disease that has significant morbidity, mortality, and healthcare burden. Glucocorticoids are the cornerstone of maintenance therapy for persistent asthma to prevent exacerbations and optional control and are recommended therapy in major guidelines [1]. Inhaled corticosteroid (ICS) improves asthma control. Subsequently, it improves the quality of life as proven in GOAL study (The Gaining Optimal Asthma Control Study), a randomized controlled trial (RCT) conducted in 2002 [2]. Both systemic and inhaled steroids are widely used in both acute exacerbations and chronic persistent asthma. They also are associated with significant side effects. Systemic steroids are associated with osteoporosis, hyperglycemia, fluid retention, and immunosuppression. ICS is associated with local effects such as oropharyngeal candidiasis and dysphonia, even though they have less systemic side effects.

Pneumonia is a condition commonly associated with chronic lung diseases such as asthma, chronic obstructive airway disease (COPD), and interstitial lung diseases. It has significant morbidity, mortality, and a global healthcare burden. In 2017, influenza and pneumonia had an age-adjusted death rate of 14.3 according to the Centers for Disease Control and Prevention (CDC) [3]. Nearly one-half (48%) of patients hospitalized with community-acquired pneumonia developed severe sepsis, and 4.5% of them developed septic shock [4]. Hence, it is important to recognize risk factors of pneumonia and patient population that are susceptible to pneumonia.

Whether ICS increases the risk of lung infections has been a matter of debate in recent years. Clinical equipoise is observed in determining the association of ICS with pneumonia, tuberculous, and non-tuberculous mycobacterial infections in both asthma and COPD patients. The evidence supporting the risk of pneumonia in COPD patients appears to be stronger [5-7]. However, fewer studies are focusing on asthma patients, and data remains controversial. In asthma patients, asthma itself is an independent risk factor for invasive pneumococcal disease [8,9]. Additionally, ICS has also been associated with mycobacterial infections in both asthma and COPD patients [10,11]. In this review, we would like to focus on the risk of pneumonia associated with ICS use in asthma patients.

## Review

Methods

The literature search was conducted on PubMed and Google Scholar by using keywords “asthma," “inhaled corticosteroids," and "pneumonia". The search was then narrowed down by using the Boolean operator "AND", as given in Table 1.

**Table 1:** Literature search results

Database	Date searched	Search term	Total studies
PubMed	January 27, 2020	Asthma	136,609
PubMed	January 27, 2020	Inhaled corticosteroids	18,740
PubMed	January 27, 2020	Asthma AND inhaled corticosteroids	11,760
PubMed	January 27, 2020	Asthma AND inhaled corticosteroids AND pneumonia	238
Google Scholar	January 28, 2020	Asthma AND inhaled corticosteroids AND pneumonia	34,100

Articles published in languages other than English and of irrelevant content such as studies including only COPD population or those on risk of infections other than pneumonia were excluded. A total of six observational studies, three RCTs, and one meta-analysis, including 86 RCTs, will be discussed in this review.

Discussion

The available evidence in literature can be divided into observational studies and RCTs. Depending on the study protocol, COPD patients were excluded in some studies but not in others. Interestingly, the risk of pneumonia varies depending on whether COPD patients were excluded and also varies among observational studies and RCTs.

Observational Studies

Observational studies conducted on the risk of pneumonia in asthma and COPD patients are given in Table 2.

**Table 2:** Observational studies conducted on the risk of pneumonia in asthma and COPD patients and their results COPD, chronic obstructive pulmonary disease; LAMA, long-acting muscarinic agonists; ICS, inhaled corticosteroids; OR, odds ratio; CI, confidence interval; HR, hazard ratio

Source	Age group	No. of patients	COPD excluded	Study type	Result	95% CI
Kim et al. [12]	>15 years	831,613	No; LAMA users included	Asthma ICS/asthma non-ICS	OR: 1.38	1.36-1.41
Ekbom et al. [13]	20-44 years	7,284	No; asthma self-reported diagnosis	Hospitalization/no-hospitalization	HR: 3.35	1.97-5.02
McKeever et al. [14]	18-80 years	43,169	Yes	Pneumonia/no pneumonia	OR: 1.24	1.5-1.79
Festic et al. [15]	43-70 years	1,234	Yes; asthma and COPD reported separately	Asthma ICS/asthma non-ICS	OR: 1.07 for asthma	0.61-1.87
					OR: 1.4 for COPD	0.95-2.09
To et al. [16]	16-83 years	62	Yes	Asthma ICS/asthma non-ICS	No difference	
Almirall et al. [17]	>14 years	2,662	Yes; asthma and COPD reported separately	Pneumonia/pneumonia (case-control)	OR: 1.1 for asthma	0.4-3.00
					OR: 3.26 for COPD	1.07-9.98

The largest retrospective observational study was conducted by Kim et al. using the Health Insurance Review and Assessment Service (HIRA) database in Korea [12]. The authors adjusted for limited confounders of age, hospital type, Charlson comorbidity index, and medical use. COPD by itself was not adjusted. They noticed an increased risk of pneumonia with the use of inhaled steroids (OR: 1.38; 95% CI: [1.46-1.41]. In the study by Ekbom et al., the asthma diagnosis was obtained through a questionnaire and a national registry of these patients for pneumonia admissions [13]. COPD patients were not excluded. They noticed an increased risk of pneumonia in asthma patients (hazard ratio [HR]: 3.35 [1.97-5.02]) more with the use of fluticasone inhaler (incidence risk ratio [IRR]: 7.92 [2.32-27.0]), and no significant association was observed with budesonide use.

In studies in which COPD patients were excluded, the risk of pneumonia was found to be not as high as in studies in which COPD patients were not excluded. McKeever et al. performed a nested case-control study using the Health Improvement Network database [14]. COPD patients were excluded in McKeever’s study. The study reported that patients with asthma and pneumonia or upper respiratory tract infections were more likely to be using steroids (OR: 1.24; 95% CI: 1.15-1.33) after adjusting for confounders. Festic et al. investigated the risk of pneumonia requiring admission with the prehospital use and after adjusting for the multiple confounding variables with logistic regression, reporting OR separately for asthma and COPD patients [15]. They concluded that prehospital ICS use was not associated with an increased risk of pneumonia in asthma or COPD patients (OR: 1.07, 95% CI: 0.61-1.87 for asthma; OR: 1.4, 95% CI: 0.95-2.09 for COPD). In a retrospective analysis of 62 patients, To et al. were not able to establish an association between ICS and pneumonia in asthma patients after excluding COPD patients [16]. Almiral et al. in a case-control study studied ICS use in asthma patients and COPD patients separately [17]. After adjusting for the confounders, they did not notice any association of ICS with pneumonia in asthma patients.

Randomized Controlled Trials

The strongest evidence comes from the retrospective analysis of the data from double-blinded clinical trials sponsored by AstraZeneca (Table 3).

**Table 3:** RCTs on the risk of pneumonia with ICS and their outcomes COPD, chronic obstructive pulmonary disease; RCT, randomized controlled trial; ICS, inhaled corticosteroids

Source	Age group	No. of patients	COPD excluded	Study duration	Study type	Result
Sheffer et al. [18]	5-66 years	7,221	Yes	3 years	Budesonide versus placebo	No difference (2.4% in the budesonide group versus 3.1% in the placebo group)
Woodcock et al. [19]	≥12 years	646	Yes	8 weeks	Five different groups of fluticasone furoate dosing versus placebo	Similar incidence to placebo in upper respiratory infection and respiratory tract infection; pneumonia not reported
Noonan et al. [20]	≥12 years	596	Yes	12 weeks	Four different groups of budesonide and/or formoterol dosing and formulation versus placebo	Pneumonia not reported
O'Byrne et al. [21]	Varies				Meta-analysis of RCT (26 trials and 60 trials)	No increased risk of pneumonia

Sheffer et al. conducted the START (Steroid Treatment As Regular Therapy) trial with 7,221 patients. They reported that budesonide does not confer any increased risk of pneumonia when compared with placebo in the three-year duration of the study (2.4% pneumonia in the budesonide group versus 3.1% in the placebo group) [18]. Woodcock et al. compared five different formulations of fluticasone furoate with placebo [19]. In the clinical trial safety outcomes, they did not report increased lower respiratory tract infections conferred to fluticasone compared with placebo. Noonan et al. conducted an RCT of 12 weeks’ duration with budesonide and formoterol formulations, but pneumonia incidence was not reported [20].

Perhaps, the best overview of RCTs on the subject can be O’Byrne et al.’s meta-analysis [21]. The study included 26 trials and a total of 14,993 patients in the primary dataset, with all trials having at least one treatment arm with budesonide and one placebo. The relative risk (RR) for pneumonia as an adverse effect was 0.52 (95% CI: 0.36-0.76; P = 0.001) and RR for pneumonia as a serious adverse effect was 1.29 (95% CI: 0.53-3.12; P = 0.58). The results were not supportive of an increased risk of pneumonia with ICS use. The secondary dataset of the study included 60 trials with 33,496 patients exposed to budesonide and 2,273 patients exposed to fluticasone and showed similar results. The meta-analysis demonstrated that the use of ICS in asthma patients has no evidence for an increased risk of pneumonia. There was no increased risk with higher doses of budesonide or any difference between budesonide and fluticasone in the trials analyzed in the secondary dataset.

Fluticasone versus Budesonide

In observational studies in which COPD patients were not excluded, fluticasone was demonstrated to have an increased risk of pneumonia compared with budesonide (Table 4).

**Table 4:** Comparison of risk of pneumonia between fluticasone and budesonide COPD, chronic obstructive pulmonary disease; CI, confidence interval; IRR, incidence risk ratio; OR, odds ratio; ICS, inhaled corticosteroids

Source	Age group	No. of patients	COPD excluded	Study type	Result	95% CI
Ekbom et al. [13]	20-44 years	7,284	No; asthma self-reported diagnosis	Hospital/no hospitalization	IRR: 7.92 for fluticasone	0.92–6.68
					IRR: 1.23 for Budesonide	1.23-4.2
McKeever et al. [14]	18-80 years	43,169	Yes	Pneumonia/no pneumonia (case-control)	OR: 1.64 for fluticasone	1.5-1.79
					OR: 1.2 for budesonide	1.06-1.35
To et al. [16]	16-83 years	62	Yes	Asthma ICS/asthma non-ICS	No difference between low-, medium-, and high-dose groups	

Ekbom et al. also showed an increased IRR of 7.92 for fluticasone (95% CI: 0.92-6.68) and IRR of 1.23 for budesonide (95% CI: 1.23-4.2) [13]. McKeever’s study excluded COPD patients and associated the use of fluticasone inhaler (OR, 1.64; 95% CI, 1.50-1.79; P = 0.001) and budesonide inhaler (OR: 1.20; 95% CI: 1.06-1.35; P = 0.003) with a higher risk of pneumonia or lower respiratory tract infection [14]. However, in To et al.’s study where COPD patients were excluded, there was no difference in pneumonia risk among low-, medium-, and high-dose groups [16]. In RCTs, there was no difference in pneumonia risk among fluticasone and budesonide [21].

## Conclusions

We noticed that the observational studies that reported an increased risk of pneumonia with ICS use did not exclude COPD. When COPD patients were excluded, observational studies did not report an increased risk of pneumonia associated with ICS use. All the clinical trials reported no increased risk conferred by ICS use with pneumonia in asthma patients. Fluticasone seems to be associated with a slightly higher risk of pneumonia in observational studies. However, the findings were not replicated in RCTs. We conclude that ICS use is not associated with increased risk of pneumonia in asthma patients and that COPD patients should be excluded in future studies concerning the risk of pneumonia in asthma patients associated with ICS use.
